# Barriers and facilitators of healthy lifestyle and perspectives towards the development of weight loss programmes. Focus groups with post-treatment breast cancer survivors in Greece

**DOI:** 10.1017/jns.2023.94

**Published:** 2023-11-03

**Authors:** Maria Perperidi, Georgios Saltaouras, Alexandros Konstandis, Marieke De Craemer, Emmanouil Saloustros, Yannis Theodorakis, Odysseas Androutsos

**Affiliations:** 1 Laboratory of Clinical Nutrition and Dietetics, Department of Nutrition and Dietetics, School of Physical Education, Sport Science and Dietetics, University of Thessaly, Trikala 42132, Greece; 2 Department of Nutritional Sciences and Dietetics, International Hellenic University, Sindos 57400, Greece; 3 Department of Rehabilitation Sciences, Ghent University, Ghent 9000, Belgium; 4 Department of Oncology, Medical School, University Hospital of Larissa, Larissa, Greece; 5 Department of Physical Education and Sport Science, School of Physical Education, Sport Science and Dietetics, University of Thessaly, Trikala, Greece

**Keywords:** Breast cancer survivors, Focus groups, Qualitative research, Thematic analysis, Weight management

## Abstract

The present study aimed to identify the factors that prohibit or enable breast cancer survivors from adopting a healthy lifestyle, as well as to record patients’ suggestions towards developing a weight-loss lifestyle intervention. Twenty-three breast cancer survivors participated in four online, semi-structured focus groups in Greece. All discussions were video-recorded and transcribed verbatim. Participants were 50⋅5 ± 7⋅4 years old with a current mean BMI of 29⋅1 ± 3⋅4 kg/m^2^. Four main themes emerged from thematic analysis: (1) dietary and lifestyle practices, (2) the effects of cancer on body weight, (3) the impact of cancer on psychology, and (4) the effect of the environment on body weight. Lack of information from healthcare professionals and lack of time were the main barriers to body weight management, whereas the main facilitators were support from their social environment, along with a comfortable physical environment, and the facility of technology. Participants suggested that an effective weight-loss lifestyle intervention should include psychological and social support, guidance and education, collaboration, flexible recommendations, personalised goals, and a follow-up plan. The needs of breast cancer survivors need to be considered when designing weight-loss lifestyle interventions. A personalised approach may prove more effective in promoting a healthy lifestyle and improving patients’ care.

## Introduction

Weight gain is common among breast cancer survivors and has been linked to disease progression, recurrence, and mortality.^(1)^ Several patients with a diagnosis of breast cancer are overweight or obese, and all women are at risk of gaining up to 5 kg in post-cancer treatment.^(2)^ Weight gain is caused by a variety of factors, including stress-related eating, decreased activity, a lowered metabolic rate due to chemotherapy, and pre- and post-therapy medications.^(3)^ Weight gain is also more common in women undergoing treatment-related menopause and is frequently accompanied by changes in body composition. This population is prone to fat accumulation and muscle loss, a condition known as sarcopenic obesity.^(1,4)^ This distinct pattern of weight gain combined with unfavourable changes in body composition is distressing for the majority of breast cancer survivors, poses a significant risk for the development of comorbid conditions, and may affect long-term disease-free survival.^(1)^

Treating obesity in breast cancer survivors is associated with higher survival rates, lower risk of metastasis, and decreased risk of developing obesity-related comorbidities, such as type 2 diabetes and cardiovascular disease. Recommendations for cancer survivors highlight the importance of treating obesity by targeting energy balance-related behaviours (nutrition and physical activity), as this may support weight loss and weight control more effectively. The most recent guidelines for cancer survivors published by the American Cancer Society emphasise the importance of counselling in providing ongoing support for survivors’ behaviour changes.^(5)^ Moreover, the World Cancer Research Fund (WCRF) recently published a guide for healthcare professionals on how to effectively support behavioural change.^(6)^ However, weight management lifestyle interventions for breast cancer survivors have not explicitly included tailored behavioural strategies in their protocols. Lifestyle interventions focusing on nutrition and physical activity changes have demonstrated effectiveness in weight loss and concomitant health benefits in several clinical trials in breast cancer survivors.^(7)^ As a result, more evidence-based nutrition and physical activity guidance following breast cancer treatment is required to help provide personalised interventions that enable survivors to adhere to the recommendations.^(8)^

Adopting a healthier dietary and physical active lifestyle with the aim to lose weight is difficult in breast cancer survivors since they face a number of challenges.^(2)^ Barriers against adopting a healthy lifestyle and obtaining healthy weight status include individual (knowledge, skills, beliefs, needs, preferences, mental health, etc.), social (e.g., family, friends, work, media, medical team), or environmental (home, time, cost, weather) factors.^(6)^ Although this information is essential to further understand the specific needs and preferences of breast cancer survivors, the current knowledge is limited. Understanding patients’ perspectives and identifying the determinants of lifestyle change and intervention delivery in breast cancer survivors is a significant step towards designing better education and communication tools to make weight loss more feasible. This may also lead to the overall development of effective, safe, well-tolerated, and equitable interventions to limit the obesity-related burden of breast cancer.^(9)^

The purpose of this study was to identify barriers and facilitators of breast cancer survivors for adopting a healthy lifestyle and record their suggestions for developing lifestyle interventions to treat overweight/obesity in this patient population.

## Materials and methods

### Ethical approval

This study was performed according to the principles of the Declaration of Helsinki and European General Data Protection Rules (GDPR) and approved by the Bioethics Committee of the Department of Nutrition and Dietetics at the University of Thessaly (07/02.02.2022). All participants were informed for the purpose and objectives of the study, signed the informed consent and provided permission for video-recording. Subsequent to their participation in the focus groups, every participant received a complimentary 60-min dietary consultation with the researcher (MP), along with a personalised diet plan.

### Participants and procedures

Participants were recruited through non-profit cancer organisations via announcements on their websites and social media during March and April 2022. Female breast cancer survivors, all over Greece, between the ages of 18 and 65, with BMI ≥25 kg/m^2^, and no active cancer therapy or ongoing treatment, except for hormonal or immune therapy, were eligible.

Breast cancer survivors were defined as women who have received a diagnosis of breast cancer and having completed overall treatment, including surgery, chemotherapy, radiotherapy, and other cancer treatments, excluding hormone or immune therapy.

A voluntary response sampling technique was used, a process that breast cancer survivors who were interested in participating in the study were invited to contact MP, who provided details on the objectives and the procedures. Prior to their assignment to the study, an online meeting was conducted between MP and every potential participant to collect information about the medical history and socio-demographic data and address any queries regarding the procedures. The study ultimately included twenty-three of the twenty-seven eligible breast cancer survivors, with the rest dropping out due to other obligations. To reach data saturation, four focus groups were conducted, each with four to nine participants. Data saturation was reached when no new themes came up in the discussion in the fourth focus group. The term ‘data saturation’ refers to the moment that no new information is added anymore on top of the already existing information. It is derived from grounded theory but is frequently used in various qualitative research designs.^(10)^

### Data collection

All focus groups were conducted with the use of an online videoconferencing platform (ZOOM). The rapid development of telehealth during the COVID-19 era can be attributed to the imposed lockdowns. However, its widespread utilisation beyond the period of confinement can be attributed to its numerous advantages, such as its efficiency in terms of time and cost. It is worth noting that telehealth does have a few disadvantages as well, primarily related to technical difficulties, specifically limited access to technology and reliable internet connections. This method was preferred because the participants were living in different areas across Greece, thus a face-to-face meeting was not feasible. Focus groups were led by MP who was the moderator and supported by GS who acted as the co-moderator (i.e. kept notes and gathered data on non-verbal expressions and body language). The researchers were registered dietitians, MP with a master's in nutrition, and GS with an MD and a PhD in nutrition and cancer. Both of them had prior experience in interviews and qualitative research. The moderator introduced the focus groups by outlining the objectives. The focus groups were semi-structured and guided using a predetermined list of topics (Table 1). The guide included questions about their knowledge of nutrition and physical activity, their previous experiences with weight management, the barriers and facilitators to weight loss, their post-cancer lifestyle modifications, and their needs/priorities for participation in a weight loss intervention. Each session lasted 60–90 min, depending on the number of participants in each group. Every meeting was videotaped and transcribed verbatim. Transcripts and video recordings were stored in a password-protected University computer in the Laboratory of Nutrition and Clinical Dietetics at the University of Thessaly.

**Table 1.** Overview of interview topics

Focus groups topics
1. Knowledge about dietary and physical activity recommendations for breast cancer survivors.
2. Body weight change experiences.
3. Barriers and facilitators in adopting healthy nutrition and physical activity and in attempting to lose body weight.
4. Current lifestyle modifications.
5. Hypothetical participation in a weight-loss intervention programme.
6. Attitudes and beliefs towards the use of digital technology for weight management.

Basic socio-demographic and medical history data were collected, including age, weight, and height; educational status; region of residence; marital status; number of children; years of survivorship; type of breast cancer; medication; and exercise level.

### Data analysis

Qualitative data were analysed using the thematic analysis approach as described by Braun and Clark.^(11)^ This method is commonly utilised in qualitative research and includes identifying, analysing, and reporting themes resulting from the data, following inductive coding. It is also considered a flexible and useful type of analysis suitable for use by non-experienced qualitative researchers.^(12)^

Prior to coding and analysing the transcripts, MP read all of them to familiarise herself with broad themes of conversations and to facilitate early identification of patterns. Then, MP and GS conducted a line-by-line coding of two randomly selected transcripts. Initial codes were then generated through discussion until a coding framework was reached. The agreed codes were applied to all focus groups, with only minor modifications. Following that, codes were organised into themes, which were later reviewed, defined, and finalised into four themes. An Excel spreadsheet was used to manage and organise data to ensure clarity, avoid losing the content of conversations, and indicate a clear justification from coding to the finalised conclusion.

Four main themes with sub-themes were identified: dietary and lifestyle practices; the effect of cancer on body weight; the impact of cancer on mental health; and the role of the environment (Table 2).

**Table 2.** The four themes and their sub-themes

Issues considering weight management in breast cancer survivors with overweight/obesity			
1. The antecedents	2. Cancer impact	3. Mental health	4. The environment
a. Knowledge	a. Body weight changes	a. Emotional state	a. Family
b. Beliefs	b. Therapies/drugs	b. Therapies/drugs	b. Social environment
c. Attitudes	c. Menopause	c. Psychology	c. Healthcare professionals
d. Motivations	d. Lifestyle changes	d. Body image	d. Natural environment
e. Expectations/unmet needs/suggestions			e. Technology
			f. Time

## Results

Participants’ demographic, anthropometric, and medical characteristics are shown in Table 3. Most of the participants had higher education level (52⋅2 %), were married (78⋅3 %), employed (69⋅6 %), and engaged in regular (at least three times per week) physical activity (60⋅9 %). Based on their medical history, most of them discovered their tumour at stage II (34⋅8 %) or stage III (39⋅1 %); most of them were positive for hormonal receptors (82⋅6 %), and most of them are currently on hormone replacement (69⋅6 %). The twenty-three participants had a mean age of 50⋅5 years, with a current mean BMI of 29⋅1 kg/m^2^, and a lower mean BMI of 26⋅9 kg/m^2^ at the time of diagnosis, based on self-reported measurements.

**Table 3.** Participants’ characteristics

Characteristics	Number (%)
Marital status	
Married	18 (78⋅3)
Divorced	3 (13)
Single	2 (8⋅7)
Education level	
Lesser (Secondary school)	5 (21⋅7)
Basic (High school, Technical school)	6 (26⋅1)
Higher (University, Masters, PhD)	12 (52⋅2)
Employment status	
Working	16 (69⋅6)
Unemployed	4 (17⋅4)
Retired	3 (13)
Stage of breast cancer	
Stage I	4 (17⋅4)
Stage II	8 (34⋅8)
Stage III	9 (39⋅1)
Unknown	2 (8⋅7)
Type of treatment	
surgery + radiation	2 (8⋅7)
surgery + chemotherapy	5 (21⋅7)
surgery + chemotherapy + radiation	16 (69⋅6)
Hormone receptors	
Positive	19 (82⋅6)
Negative	3 (13)
Unknown	1 (4⋅4)
Hormone therapy	16 (69⋅6)
Physical activity	
Regular physically active	14 (60⋅9)
Occasional physically active	2 (8⋅7)
Sedentary	7 (30⋅4)
Years of survival*	5⋅8 ± 5⋅2
Age (years)*	50⋅5 ± 7⋅4
Weight at diagnosis (kg)*	69⋅7 ± 10⋅1
Current weight (kg)*	75⋅6 ± 8⋅7
Height (cm)*	161⋅3 ± 5⋅4
BMI at diagnosis (kg/m^2^)*	26⋅9 ± 4⋅8
Current BMI (kg/m^2^)*	29⋅1 ± 3⋅4

* Data presented as mean ± standard deviation.

### Dietary and lifestyle practices (antecedent conditions)

The majority of the participants stated that they acknowledged what ‘healthy’ or ‘unhealthy’ nutrition meant, as well as what foods to eat for a healthy lifestyle.

- ‘*I know that I must consume foods from all food groups; I know it's bad, but I just love sweets. I lived on sweets’ P920*.
- *‘I am aware that “bad” nutrition is harmful; I am aware of this because I have read far too many articles’ P601*.

Most of the participants have never heard of or been informed about breast cancer survivors’ lifestyle recommendations, except for two who were told by their oncologist to adopt the Mediterranean diet, two others whose oncologist told them to watch their weight, and three more who consulted a dietitian by themselves or were informed by a non-profit breast cancer survivors organisation.

- ‘*Speaking personally, officially from my doctor, my oncologist, I never received any specific recommendation, any guidance, or anything’ P702*.
- *‘My doctor suggested I follow the Mediterranean diet, reduce red meat consumption, eat a lot of fruits and vegetables, choose no other oils except olive oil and avocado, and avoid processed foods. That's what he recommended’ P610*.

Obesity and diet were considered negative concepts for the majority of participants. Their perceptions of obesity include its characterisation as a disease or bad health, with a link to cancer in general, comorbidities, and negative emotions. Additionally, diet was mainly described as ‘*deprivation*’, ‘*programme*’, ‘*restriction*’, ‘*desperation*’, or ‘*a painful story*’ with only three participants believing that diet is a healthy way of eating.

- ‘*I have never linked obesity to cancer, but rather to other diseases like diabetes or heart disease’ P608*.
- *‘For me, obesity is definitely a disease. For me, it has a significant and direct impact on my mental health’ P919*.
- *‘A diet means deprivation for me; I will have to give up some of the things that I currently enjoy’ P203*.

Regarding their attitudes towards lifestyle, nutrition, and weight control, participants admitted that weight loss was often their objective before cancer diagnosis, which was achieved through healthy nutrition and lifestyle in general.

- ‘*I may not have been obese; I was overweight, but I have always been concerned about diets, either with dieticians or on my own’ P812*.

All participants wanted to change their lifestyle, and their motivations included being physically and mentally healthy, energetic, having overall wellness, improving their appearance, loving themselves, practicing self-care, avoiding recurrences, and being alive.

- ‘*Yes, I definitely want to lose weight. I don't prioritize health, maybe because my experience with cancer is more recent; I want it only for aesthetics and appearance’ P203*.
- *‘I just want to lose my extra body weight because I've read research that says extra body fat increases the chances of relapse, and I don't want to go through that again, or at least avoid it for a long time’ P610*.
- *‘That's enough. I think I must start over. I am a young woman; I can't feel so bad; everything hurts. We must survive’ P601*.

Based on their previous experiences, all participants expressed their needs in terms of body weight control. These unmet needs included emotional support, cooperation, education, flexibility in recommendations, individualised goals, demarcation but not deprivation, persistence in maintenance, and lifetime changes.

- ‘*I have a complaint: in my opinion, there should have been recommendations and support for women going through this’ P702*.
- ‘*Group sessions have many advantages. It will not, however, be able to meet individual needs. So I prefer a mix of group and individual sessions because we don't all have the same needs, ailments, or experiences’ P1021.*
- *‘I think I should do it for the rest of my life. I*’*m overweight, and I have to fight it. I believe I will achieve this through maintaining contact’ P1022.*
- *‘I don*’*t want to deprive myself of that. We will fix the body, and we will destroy the psychology. I believe in everything in moderation’ P615*.

Moreover, participants had several expectations and suggestions about weight management programmes and interventions.

- ‘*A diet plan adapted to everyone's special needs; to be flexible, with some suggestions. I prefer the group session, where we can talk about what worries us, what obstacles we face, and what went well’ P702*.
- *‘For me, the ideal is a combination, because individual sessions may provide more targeted suggestions, but group sessions provide better communication and support. We all struggle together’ P1021*.

### Effects of cancer on body weight

All participants stated that they gained weight after completing their cancer treatments, except for one (P1022). The majority of them agreed that losing weight after cancer treatments was extremely difficult and that they needed to adjust to their new metabolism. Cancer treatments, drugs, hormone therapy, and menopause were the primary causes of their excess body weight, according to all breast cancer survivors. Some of the women were diagnosed at the beginning of menopause, and the premenopausal breast cancer survivors should undergo artificial menopause. In any case, the participants would have to deal with their cancer as well as menopause, which are both difficult conditions. Aside from menopause, some of the participants believed they gained weight as a result of their unhealthy lifestyle.

- ‘*After breast cancer treatments, my hormone profile changed, and I gained weight that I can't get rid off’ P514*.
- *‘I don't believe I gained weight solely because of the treatments, menopause, or diet. These reasons are not sufficient. It's our fault as well; perhaps we ate more; I ate more than I should have’ P615*.

The majority of participants tried to manage their body weight after cancer treatments, thus altering their lifestyle, either through dietary changes, physical activity changes, or both. In very few cases, cancer or its treatments led to poorer dietary habits.

- ‘*I cut back on my dinner portion and no longer add sugar to my coffee. I also do Pilates twice a week and walk on a regular basis’ P304*.
- *‘I changed my lifestyle. I have a daily routine that I'm trying to stick to. Only through this program I was able to lose my excess body weight. I improved my diet and increased my physical activity; it was a huge and painful effort, but the final result was well worth it. I'm absolutely pleased’ P608*.

### Impact of cancer on psychological and body weight status

The emotional state of participants after completing cancer treatments was a major topic of discussion in all focus groups. It emerged that experiencing feelings such as stress, sadness, depression, and anger pushed the breast cancer survivors to engage in more unhealthy behaviours and become trapped in a vicious cycle of negative thoughts like disappointment, guilt, and being emotionally upset, reaction, denial, and/or exhaustion. Calmness and joy, on the other hand, led in some cases to self-care.

- ‘*Because of my stress, I ate more’ P610*.
- *‘To love myself and to take care of myself also helped me deal with cancer and its treatments’ P113*.

The majority of breast cancer survivors believed that hormone therapy and anti-menstrual medications could have a negative impact on their emotional health as well as their body weight. Two women stated that after completing cancer treatments, they felt depressed and required antidepressant medication, which adversely affected their body weight.

- ‘*I am unable to control my appetite because my hormone therapy has also had an emotional impact on me’ P702*.

All participants discussed the role of their mental wellbeing in changing their mindset. As a result, mental wellbeing appeared to be both a barrier and a facilitator of weight management. Additionally, weight control success appears to lead to psychological improvements in breast cancer survivors.

- ‘*I don't feel psychologically well enough to stick to the diet prescribed by the dietitian’ P601*.
- *‘Diet is a psychological issue for me. When my mental wellbeing is strong, when I feel good, it helps me avoid eating unhealthy foods’ P920*.

Body image and feelings about body changes were frequent topics of discussion among focus groups participants. Breast cancer survivors stated that changes in body image can strongly influence participants’ psychology and behaviours.

- ‘*I am bothered by my current image. I want my previous body image back. This is too intense for me’ P615*.
- *‘I'm angry that I've gotten to this point of being obese. I'm very angry. Why should I gain so much weight?’ P615*.

### Effect of the environment

The family's role in weight loss was thoroughly discussed. It was helpful in some cases and hindering in others.

- ‘*They were extremely supportive. My husband in particular, but also my parents and the rest of the family. I couldn't do anything without them, especially my husband. 90 % of my effort was accounted for by his psychological support’ P316*.
- *‘I got divorced and gave up everything. Unfortunately, I had given up on myself’ P601*.

Aside from family, the social environment appeared to be important in weight management, either as a barrier or as a facilitator.

- ‘*The obstacle is that on the weekends I go out with friends, and I want to enjoy it, so I eat and drink without restriction’ P608*.

Support from healthcare professionals was a major issue for breast cancer survivors. The focus groups revealed that participants expected information, education, recommendations, and support from their medical team, particularly their oncologist.

- ‘*No doctor deals with nutrition; no doctor tells you to change your way of life’ P113*.

Natural environmental conditions have been found to influence participants’ weight loss efforts in a variety of ways. The coronavirus pandemic and impending confinements, as well as weather and home environments (food choices, gym equipment), all had an impact on outcomes, both positively and negatively.

- ‘*When I locked myself up at home, I gained weight again’ P608*.
- *‘The nice weather helped me go for a walk’ P609*.

The vast majority of participants stated that they were familiar with digital technology or that it could be adapted. Most of them found technology a facilitator in their efforts to change their behaviours. Among other things, they could save time and eliminate distance by using digital media.

- ‘*Technology does not bother me. Every one of us is incorporating online communication into our daily lives. So, the distance will not be an issue’ P1021*.

Finally, time management could either aid or inhibit participants’ efforts to change their lifestyle.

- ‘*I do too many things, I don't have enough time, I get tired, and I eat. The nibble is my way out’ P702*.

## Discussion

This study explores breast cancer survivors’ beliefs, experiences, barriers, and facilitators about weight management and provides an in-depth perspective about their needs and expectations of a weight-loss intervention programme. According to the findings, the majority of the participants were not informed about the lifestyle changes they should make after cancer or about the recommendations of cancer survivors. Their main motivation for making lifestyle changes was to maintain their health. Most of them had struggled with weight control issues prior to cancer, and they had tried a variety of dietary patterns; resulting in their attitude towards diet being negative. They did not link breast cancer to their excess weight, but somehow they changed their dietary and physical activity habits after being diagnosed. All except one participant gained weight after cancer treatments, believing that it was primarily due to side effects of cancer medications and therapies, menopause, and/or post-cancer hormone therapy. A vicious cycle was observed in which post-cancer body changes affected body image, impacted psychological status, influenced dietary choices, and closed the cycle with an impact on body weight. Barriers like negative psychology, temptations, a lack of information, and a lack of time, as well as facilitators like positive psychology, support from family, medical team and social environment, good time management, and easier environmental and technological conditions, were identified. Lack of family support appeared to be an important determinant for the adoption of a healthy lifestyle among the participants. Finally, based on their previous experiences, breast cancer survivors expressed their needs and expectations of a weight-loss intervention programme that included psychological support, counselling and education from credible healthcare professionals, collaboration among specialists, recommendations that can be adjusted to their needs, avoidance of restrictions, individually tailored goals, and a long-term weight maintenance plan.

The absence of information about post-cancer lifestyle recommendations observed in this study is an issue that systematically appears throughout the survivorship care trajectory. According to an Australian study on weight management after breast cancer, 79⋅79 % of women reported not receiving any advice about weight loss or weight gain prevention at the time of diagnosis, and researchers declared significant information gaps regarding weight management in this population.^(13,14)^ These findings were confirmed by similar studies in Ireland, Malaysia, and China.^(15–17)^ Reviews of qualitative studies reported ineffective dietary guidance and limited weight control follow-up from healthcare professionals.^(18–21)^ All these studies also highlighted the critical role of health professionals in monitoring weight, providing advice, and referring to a multidisciplinary team. The contradictory messages in dietary advice and weight management following cancer have also been noted.^(20,22)^ This lack of communication with healthcare professionals could be linked to a lack of willingness among breast cancer survivors to adopt healthier lifestyles.^(23)^ It has been shown that a healthcare professional’s recommendation to exercise significantly improves physical activity engagement and weight management.^(7)^

The identification and reinforcement of motivations are critical for achieving behavioural changes. Several studies have revealed that a lack of motivation is a significant impediment to adopting new lifestyle habits.^(13,15,19)^ Our study showed that participants’ key motivators for change was maintaining physical and mental health, along with improving their appearance. Previous studies have also identified healthy eating to prevent recurrences as a motivator.^(14,17)^ The increase in aesthetic evaluation to improve self-acceptance was a primary motivation for breast cancer survivors to participate in a behavioural intervention. The same study found numerous other motivations, including participants’ physical and mental health, coping with the illness, and sharing their experiences.^(24)^ Motivations should also be enhanced because they facilitate intervention adherence.^(25)^ For this purpose, motivational interviewing may be useful in behavioural interventions. This behaviour change technique is effective in determining and exploring motivations, overcoming hesitation, and reinforcing behaviour change.^(15)^

The willingness to change an individual's lifestyle is greatly influenced by personal beliefs, attitudes, and thoughts about diet, body weight, and their connections to cancer. Most of the participants in this study had negative attitudes towards diet as a result of pre-cancer restrictive dietary practices, and they did not correlate their diagnosis with their excess body weight, which could be a barrier to behavioural modifications. Earlier studies indicate that the most frequently mentioned personal perceptions as impediments to lifestyle change are beliefs that cancer is unrelated to lifestyle and uncertainty about the benefits of lifestyle in relation to cancer and health.^(16,19)^ Female cancer survivors held ambivalent beliefs about the relationship between body weight, dietary intake, and physical activity to their cancer diagnosis and potential recurrence.^(26)^ Personal beliefs and preferences regarding diet–cancer relationships have been described as a risk factor for post-treatment dietary information needs.^(18)^

All but one participant in this study admitted to gaining weight after being diagnosed with cancer. The only participant who lost weight had morbid obesity, and her doctors advised her to lose weight as a part of her cancer treatment. An Australian study found that 64 % of participants gained weight overall after diagnosis, with an average weight gain of 9⋅1 kg.^(13)^ Many other studies have confirmed weight gain from treatments and premature menopause after breast cancer diagnosis.^(14,22,26)^ Cancer-induced menopause is a major contributor to weight gain following breast cancer.^(3,27,28)^ Other causes of weight gain have been identified, including ‘stress eating’, decreased physical activity, chemotherapy-induced lower metabolic rate, and cancer medication use.^(3)^ Evidence from the Women’s Healthy Eating and Living (WHEL) study reported that chemotherapy was significantly associated with weight gain, but hormone therapy (Tamoxifen) was not. More specifically, women who received chemotherapy were 65 % more likely to have gained significant weight than women who did not receive either chemotherapy or hormone therapy.^(28)^ Many women attribute their weight gain to hormonal therapies such as Tamoxifen.^(14)^ Tamoxifen may exacerbate the body composition changes that occur with the onset of menopause.^(29)^

The psychological impact of cancer on participants’ body weight was discussed thoroughly, and weight cycling was observed as a vicious cycle of negative emotions, impacted body image and weight gain. In other words, this could be the vicious cycle of emotional hunger. As mentioned previously, in the current study, participants’ impaired body image triggered emotions such as stress, sadness, depression, and anger, which drove them to engage in unhealthy behaviours, which trapped them in disappointment, guilt, emotional upset, and other negative thoughts, leading them to more unhealthy behaviours. Breast cancer survivors suffer from severe psychological distress after gaining post-treatment weight.^(30)^ The overall sense of bodily self, including body image, could be damaged after diagnosis and treatments.^(24)^ According to emerging evidence, negative body- and weight-related emotions are important and often overlooked predictors of psychological health. In particular, shame and guilt have been identified as important body-related emotions in individuals struggling with weight and may be pertinent relevant in the experience of breast cancer.^(30)^ A recent meta-synthesis about body image experiences of women with breast cancer concluded that women’s body image and its psychological implications should be considered a process in constant development.^(31)^ For these reasons, strategies to ameliorate these negative feelings are essential for women with breast cancer. Physical activity may be one of them, as they promote positive body image, emotional regulation, cognitive development, and individual self-esteem.^(32)^ The grounded theory of body image for women diagnosed with breast cancer should be taken into consideration as it illustrates intrapersonal and interpersonal factors and strategies to manage their body image.^(33)^

This study identified the main obstacles in managing weight after breast cancer, which included negative mental wellbeing, temptations, a lack of information, and a lack of time, as well as the main enablers, which included positive psychology, support from the family, the medical team and the social environment, good time management, and more conductive environmental and technological conditions. Only a few previous studies have explored barriers and facilitators of weight management in breast cancer survivors. An Australian study recorded fatigue among weight loss barriers and identified a structured exercise programme, a prescribed diet, accountability to someone else, and social support among the facilitators.^(13)^ In the previous study, only 4⋅3 % of breast cancer survivors thought a breast cancer-specific programme would be beneficial. A more recent study by Ee *et al.* included specific advice from healthcare professionals, environmental restructuring (including financial support), motivations (creating an expectation of looking and feeling better), persuasion (aiming to prevent recurrence), and providing women with specific knowledge and skills as major enablers in adopting optimal lifestyle behaviours and weight management.^(14)^ Another study identified the lack of individualisation, lack of time, and lack of support as important barriers, but knowledge on food preparation and recipes, goal setting, a stable routine, and support from friends and family as facilitators for diet interventions.^(25)^ The lack of information or advice from healthcare professionals, the lack of time, and the role of family, and social support in weight management have been thoroughly explored by many other studies in breast cancer survivors.^(15,17,22,34,35)^

Finally, participants discussed their needs and expectations for a weight-loss intervention programme. Breast cancer survivors stated that psychological support, guidance, and education from reputable healthcare professionals, collaboration among specialists, flexibility in recommendations, avoiding restrictions, individually tailored gradual goals, and a long-term weight maintenance plan could help them control their weight more successfully. These findings are in accordance with a scoping review, where cancer survivors preferred specific recommendations, direct interaction with the medical team, and peer support from other cancer survivors.^(20)^ Face-to-face communication, online and telephone support, printed materials, and different specialists to share care in the primary healthcare setting were among the preferences of cancer survivors for the delivery of dietary information after treatment.^(18)^ On the other hand, group interactions facilitate strategic skills and encourage self-reflection and potential change, thus may be beneficial to share feelings and experiences with others who have experienced a similar position.^(32)^ According to a survey of 122 breast cancer survivors, the respondents indicated that they preferred virtual platforms to in-person meetings (68⋅9–21⋅3 %), due to better time management.^(23)^ The use of technology could facilitate the participation in weight-loss intervention programmes.

### Strengths and limitations

The findings of the present study should be considered under the light of its strengths and limitations. To our knowledge, this is the first study to investigate the needs and perceptions of breast cancer survivors prior to a weight-loss lifestyle intervention in Greece. Focus groups were conducted by an online videoconferencing platform, which has the advantages of convenience, time efficiency, and cost-effectiveness, while also facilitated the participation of individuals residing in geographically distant areas. However, it is important to acknowledge that this approach has certain limitations, including technical difficulties and the potential for a partial loss of non-verbal information, particularly body language rather than facial expressions. Additionally, sampling and selection bias may have occurred. Subjects were recruited through self-selection via announcements on cancer non-profit organisations’ websites and social media, and those who were well-educated, most keen on digital technology, most motivated, and most interested in nutrition and health were more likely to participate. All data, including body weight and height measurements, were self-reported, making it vulnerable to recall bias and socially desirable answers. Finally, no qualitative data analysis software was used, and thus, researcher bias may have emerged, despite the fact that the qualitative analysis was closely monitored by two authors (MP and GS).

## Conclusions

This study identified barriers and facilitators, and it also revealed unmet weight management needs among breast cancer survivors in Greece. The impact of psychology on weight control was assessed. The main barriers were a lack of information, time, and family support while the main facilitators were support from family, friends, and healthcare professionals, as well as easier environmental and technological conditions. According to participants, a weight-loss lifestyle intervention would have been more effective if it included psychological and social support; counselling and education from credible healthcare professionals; specialists’ collaboration; flexibility in recommendations; avoidance of restrictions; personalised, gradual goals; and a long-term weight maintenance plan. Identifying the unmet needs of breast cancer survivors could be supportive to healthcare professionals to design more effective weight-loss lifestyle interventions for breast cancer survivors.
